# TEOS Nanocomposites for the Consolidation of Carbonate Stone: The Effect of Nano-HAp and Nano-SiO_2_ Modifiers

**DOI:** 10.3390/ma15030981

**Published:** 2022-01-27

**Authors:** Alexandra Rodrigues, Bruno Sena da Fonseca, Ana Paula Ferreira Pinto, Susana Piçarra, Maria de Fátima Montemor

**Affiliations:** 1 Escola Superior de Tecnologia do Barreiro, Instituto Politécnico de Setúbal, R. Américo da Silva Marinho, 2839-001 Lavradio, Portugal; susana.picarra@estbarreiro.ips.pt; 2 Centro de Química Estrutural-CQE, Department of Electrochemistry (DEQ), Instituto Superior Técnico, Universidade de Lisboa, Av. Rovisco Pais 1, 1049-001 Lisboa, Portugal; bruno.fonseca@tecnico.ulisboa.pt (B.S.d.F.); mfmontemor@ist.utl.pt (M.d.F.M.); 3 CERIS, Department of Civil Engineering, Architecture and Georesources, Instituto Superior Técnico, Universidade de Lisboa, 1049-001 Lisboa, Portugal; anapinto@civil.ist.utl.pt

**Keywords:** consolidation, carbonate stone, TEOS nanocomposites, hydroxyapatite nano-rods, silica nanoparticles

## Abstract

This study aimed at evaluating the effect of hydroxyapatite (HAp) nanosized structures and nanoparticles of hydrophilic silica as modifiers of both acid- and alkaline-catalysed tetraethoxysilane (TEOS)-based products for the consolidation of carbonate stones. Their initial effectiveness and some compatibility aspects were assessed in a porous limestone (sound and artificially aged Ançã stone samples) and two types of treatment (capillary absorption and brushing). The studied products were examined by scanning electron microscopy (SEM) and micro-Raman spectroscopy. Their depth of penetration and strengthening effect were evaluated through drilling resistance. Their action on the substrate was also further assessed by non-destructive methods based on colour variation and Shore-D hardness. Treated stone samples were dissimilarly affected by the tested treatments and exhibited a significant increase in strength with a low risk of over-strengthening. Adequate in-depth penetration patterns, as well as colour compatibility with the substrate were obtained with some of the prepared formulations through two types of treatment, both in sound and aged stone samples. The potential most effective treatments with the lowest colour change were obtained with the acid-catalysed TEOS-based products modified with HAp nanosized structures.

## 1. Introduction

Carbonate sedimentary stones are a well known and highly representative group of materials in cultural heritage, particularly built heritage. Despite being historically widely applied (e.g., Portuguese Ançã stone, Maltese Globigerina stone, Dutch Maastricht stone, French Tuffeau stone, among others) [1,2,3,4], high porosity limestones are sensitive to outdoor environments, suffering several decay processes that may impair their integrity [1,5]. These materials often demand active conservation actions to prevent or slow down material losses [6]. Consolidation of decayed carbonate stone has been intensively studied to find appropriate treatments to address the fragile surfaces of these stones (e.g., [7,8,9]). Due to compositional and textural complexities, positive results consequent of such treatments are still scarce [8].

Presently, alkoxysilane-based products are still the most well-established and applied stone consolidants, due to recognised advantages, such as: (i) low viscosity, allowing for in-depth penetration into the stone pores, (ii) versatility and the possibility of modification and adaptation to a wide range of stone substrates, as to other porous materials, (iii) practical and easy application, and (iv) resulting in inert products with chemical stability (photochemical, thermal, etc.), among others [6,10,11]. The formulation of suitable alkoxysilane-based products is not simple since sol–gel reactions and the characteristics of the resulting consolidating material are very sensitive to a wide range of variables (e.g.,pH, type of catalyst, solvent nature and its relative amount, water content and type of precursors, incorporation of organic components, etc.) [6,10], whereas the final characteristics of the consolidating material influence critical features of the treated carbonated stone (e.g., hardness or colour) [6,12]. Other solutions especially developed for the consolidation of carbonate stones are also worth noting, showing several potentialities. Examples include the nanolimes, the oxalate-inorganic-salt-based solutions, or the diammonium hydrogen phosphate ones, besides the alternative treatments based on biomineralisation of CaCO_3_ by microbiota (e.g., [9,13,14,15,16]). When all these products are used in situ, multiple results can be obtained (e.g., [1,9,17]), and many variables need to be considered for the potential success in each case, since their performance may depend on slightly varying reactional parameters or application procedures. Therefore, as exemplified by the several studies on consolidation materials aforementioned, research on this subject is still very actively ongoing and in need of further development.

Nanomaterials science has been considered an interesting alternative route to enable new solutions in the stone conservation field [18,19]. Thus, the modification of the predominant alkoxysilane-based or TEOS-based formulations for stone consolidation has been widely attempted (e.g., [20,21,22]). The development of nanocomposites based on colloidal dispersion of nanoparticles showed promising advantages (e.g., penetration depth, increase of the specific surface available to the reactions) [20,21]. Some nano-modifiers were mainly applied for other purposes—nano-TiO_2_, -SnO_2_, or -Al_2_O_3_ biocide and/or self-cleaning properties (e.g., [23,24,25,26]) TEOS-based particle-modified consolidants with a noticeably reduced degree of cracking have been often reported [27,28,29,30,31], for instance with the use of silica nanoparticles. Besides, calcium phosphates, including hydroxyapatite (HAp), were found to deliver some interesting results to carbonate stones and seem particularly suitable for stone reinforcement and weathering resistance (e.g., [32,33,34,35]). Nevertheless, only occasionally, HAp nanoparticles have been used to modify TEOS-based formulations [36,37]. Their beneficial effect seems to extend from spherically shaped [36] to tuned high-aspect-ratio nano-HAp, the latter possibly offering even more promising results [38].

The development of new or improved solutions for the consolidation of carbonate sedimentary stones is of great relevance for contemporary scientific disciplines such as conservation, civil engineering, geology, materials science, among others. Hence, this paper aims at evaluating the potential improvement of the properties and consequent performance of TEOS-based formulations by the addition of: (i) hydroxyapatite nanorods (HAp), recently tested and showing promising results [38]; (ii) hydrophilic silica (SiO_2_) nanoparticles, more commonly applied. Typically, nanosilica seems to have been applied in stone consolidation in its originally hydrophilic form (see, for instance, [29,39,40] and the references therein).

The role of these two modifiers and their improvement potential compared to the non-modified (reference) formulations were analysed. Additionally, a comparison was made between the novel products obtained by modification with (i) and (ii) in two different media: acid and alkaline TEOS-based formulations. This assessment focused on a critical comparison of these formulations applied on a soft carbonate stone used in built heritage. Analytical methods were employed to determine the potential of these nanocomposites to restore stone cohesion, penetration patterns, and compatibility with the substrate. Effectiveness, as a measure of the strengthening effect of a consolidation treatment, and compatibility, as a measure of “harmony” or “similar behaviour” for the substrate [1], were evaluated.

To widen the range of scenarios studied, two types of treatments were assessed: capillary absorption and brushing on a selection of the best-performing formulations. The results of brushing treatments on sound and aged stones were also compared. The aim was to contribute to the knowledge on the variables studied in terms of the potential initial efficacy of the modified products and on any incompatibility risks that may exist related to possible hardened layers and colour alterations.

## 2. Experimental Section

### 2.1. Reagents

The reagents used for the formulation of the sols were: tetraethyl orthosilicate (TEOS) as a precursor, ethanol at 97% (EtOH) as a solvent, and distilled water for the hydrolysis reactions. As basic catalysts, (i) ammonia solution 25% (NH_3_) and (ii) octylamine (Oct) were used, and as an acid catalyst, HCl was used. As an additive flexible organic chain, a low-molecular-weight grade of poly(ethylene glycol) (C_2n_H_4n+2_O_n+1_, where n=8.2 to 9.1, PEG_400_, $M_{w}\approx 400$ g·mol^−1^) was used. The calcium carbonate powder used was a finely ground Ph Eur.-precipitated salt with 96% CaCO_3_. All these reagents were purchased from Sigma-Aldrich and used without further purification.

The modifier agent nano-hydroxyapatite (nano-HAp) was prepared with a diameter around 5–20 nm and a length up to 125 nm, according to a recently developed method [38]. The hydrophilic silica nano-particles (nano-SiO_2_) were from Wacker (HDK^®^ T40 hydrophilic pyrogenic silica).

### 2.2. Stone Samples

The carbonate stone used for testing the sols (formulations) was the “Ançã stone” type, a very homogeneous and soft limestone that has an accessible porosity of around 27%, a coefficient of water absorption by capillarity of around 150 g·m${}^{-2}$s${}^{0.5}$, and a pore size range from 0.1–1 μm, as reported in previous applications [41,42]. This stone is soft and decay-susceptible [1].

Sound Ançã stone samples ($30\times 30\times 30\;{\mathrm{mm}}^{3}$) were used for treatment, as well as similar-sized aged Ançã stone samples (submitted to ageing cycles of 24 h freeze–thaw, with 7±1 h freeze, as described elsewhere [43]). Examples are shown in Appendix A.

### 2.3. Preparation of Sols

Sols (formulations) were prepared by magnetic stirring of TEOS and EtOH (or EtOH + HAp where applicable) for 30 min (≈550 rpm) (molar ratios of 1:7.6 TEOS:EtOH), followed by the addition of distilled water and one of the catalysts (NH_3_, Oct, HCl). Upon the addition of water and one catalyst, two main sol–gel routes were tested: acid (HCl) and alkaline (NH_3_ or Oct).

Two acid-catalysed formulations were prepared: (i) one where HCl was added and kept under magnetic stirring (≈550 rpm) for 2 h in a closed flask at room temperature (24 ± 2 °C)—see a similar approach, for instance, in [44,45]—and (ii) a second one where the above-described procedure was followed by the addition of PEG under stirring for 1 min (TEOS:PEG 1:0.11 molar ratio).

Two base-catalysed formulations were also prepared: in each, one of the two alkaline catalysts (Oct and NH_3_) was added, and the mixture was kept under magnetic stirring (≈550 rpm) for another 30 min in a closed flask, in the same conditions as the acid formulations (for a similar approach, for instance, see [46,47]).

The TEOS:H_2_O molar ratio was 1:1.5 for basic catalysis and 1:2.1 for acid catalysis. The TEOS:“catalyst” molar ratios were kept constant (1TEOS:0.002Oct, 1TEOS:0.03NH_3_, and pH’ = 3.2 upon the addition of HCl)—see Table 1.

The addition of nano-SiO_2_ to the respective formulations was performed at the end of the aforementioned stirring time (after PEG addition, when applicable), and magnetic stirring was kept for one more minute. For nano-HAp-modified formulations, the preparation was preceded by a dispersion of the nano-structures in the solvent EtOH, for 1 h under sonication, as in Rodrigues et al. [38]. In all cases, the formulations were sonicated for 1.5 min after all reagents had been added and stirred.

The resulting materials are denoted by catalyst, catalyst:nHAp, or catalyst:nSiO_2_, whenever nano-HAp or nano-SiO_2_ was added—i.e., for instance, “HCl:PEG” where neither nano-HAp, nor nano-SiO_2_ were added and “HCl:PEG:nSiO_2_” when nano-SiO_2_ was added to the sol. The molar ratios of the mixtures are summarised in Table 1. The amount of nano-additives added was always in the proportion of 1:0.01 TEOS:*additive*. This amount was set for comparison with the literature using HAp nanoparticles [36].

The preparation of sols was replicated for the three drying environments, as well as for the sound and aged applications by capillary and brushing procedures, described in the following sections.

### 2.4. Drying Environments

Three procedures were adapted according to previous studies [45,47,48], representing three different drying environments designed for the study and characterisation of the xerogels, the carbonate environment, and the stone pore system:

(a) Fifteen millilitres of the formulations were placed inside cylindrical flasks (3.5 cm diameter) with pinholes (needle-size) in the cap (xerogels);
(b) Ten millilitres of the formulations were mixed with 10 g of the CaCO_3_ powder and placed inside cylindrical flasks (calcite blends);
(c) The formulations were applied to sound and aged limestone samples ($30\times 30\times 30\;{\mathrm{mm}}^{3}$) by two different procedures: capillary suction and brushing. Specifications are given in Section 2.6.1.

The resulting materials were kept in a chamber at 20±5 °C and 60±10% relative humidity (RH).

### 2.5. Characterisation of the Formulations

An early screening process, which included the observation of the homogeneity, density, measurement of viscosity, evaluation of the apparent gelling time (macroscopic observation), and cracking tendency of the xerogels, was determinant for assessing the formulations’ viability [49]. The viscosity of the sols was measured with a sine-wave vibro-viscometer SV-10 with the range of measurement from 0.3 mPa s to 10,000 mPa·s. The stability of the xerogels and calcite blends was assessed over time using weight measurements, until the weight changes were <0.01 g over 7 d. Xerogels were characterised at the chemical and microstructural level, respectively by: Raman spectroscopy, where a confocal high-resolution micro-Raman spectrometer LabRAM HR 800 Evolution (200–1600 nm), with an external diode laser of 532 nm, was used, and SEM with a Carl Zeiss AURIGA CrossBeam FIB-SEM coupled with an Oxford INCA Energy 350 EDS detector.

The cohesion ability of the sol was assessed through: (i) the determination of the cohesion class of calcite blends—by manually breaking after stabilisation—and (ii) Shore Hardness A measurements.

Using the expedite assessment (i), the relative resistance was established among 0 (no cohesion), 1 (break by handling), 2 (easy to break), 2.5 (not so easy to break), 3 (hard to break), and 4 (very hard to break manually), equivalent to classes elsewhere used from (−) up to (++++) [42,44,45,47].

For the method (ii), a Shore durometer type A (LCD Display Meter 0–100HD) with a standardised presser foot—i.e., a hardened steel rod (SR) of 1.1–1.4 mm in diameter, with a truncated 35° cone, and a 0.79 mm diameter truncated tip—was used to measure the depth of indentation in the material to a given force of 8.1 N. The resulting value registered (between 0 HD and 100 HD) corresponds to the resistance of the material to indentation.

### 2.6. Efficacy

#### 2.6.1. Treated Stone Samples

Sound Ançã stone samples were treated with all the studied formulations by capillary suction for 3 h. The key parameters influencing the performance of the treatment were firstly assessed during the application of the formulations, specifically, the capillary fringe evolution—indicative of the penetration ability within the porous system—the absorbed product (variation of weight after 3 h of capillary suction), stabilisation time, and dry residue (variation of weight over time).

A set of formulations, selected based on the initial characterisation performed on the samples treated by capillarity, was applied onto sound and aged Ançã stone samples by brushing. This treatment followed the criteria of application until apparent refusal, a similar procedure to the one that usually is performed in real situations [50,51]. The absorbed product, stabilisation time (monitored by measuring weight loss over time), and dry residue were registered. Weight remained stable in all treated samples after about 2 months, and the following tests were carried out.

#### 2.6.2. Consolidating Effect

The initial consolidating effectiveness of selected formulations was evaluated by the (i) hardness increments caused on surfaces and (ii) drilling resistance across the sample’s depth.

(i) Shore Hardness D measurements (minimal sampling of six measurements per stone surface) were performed with a Shore D durometer (LCD Display Meter 0–100 HD) with a standardised indenting foot—hardened steel rod with a 30° cone and a 0.1 mm radius tip—that measures the depth of indentation in the material to a given force of 44.5 N. When the foot penetrates the surface of the material, the resistance of the material to indentation is registered between 0 HD and 100 HD.

(ii) A drilling resistance measurement system (DRMS) was used to perform DR measurements across the samples’ depth. To minimise the disturbance of the data by stone powder packing, a guide hole with a 3 mm diameter was performed across the 30 mm samples. The test was made by using a 5 mm diameter diamond drill bit to drill over the guide hole, and the resultant powder was vacuumed through the opposite side of the guide hole during the drilling. All drilling tests (minimal sampling of three holes per stone sample) were performed at a speed of 100 r.p.m. and at a 20 mm/min penetration rate.

### 2.7. Colour Variation

The colour variation of the limestone was investigated as one of the diverse compatibility indicators that needs to be considered. Total colour variation ($\Delta E^{*}$) was assessed with a colorimeter KangGuang WSD-3A. The colourimetric coordinates ($L^{*}$, $a^{*}$, $b^{*}$) were registered (minimal of five measurements per surface) before and after treatment (e.g., $\Delta L^{*}=L_{after}^{*}-L_{before}^{*}$). The difference between the average values was calculated by $\Delta E^{*}$, as follows:

(1) $$\Delta E^{*}=\sqrt{{\left(\Delta L^{*}\right)}^{2}+{\left(\Delta a^{*}\right)}^{2}+{\left(\Delta b^{*}\right)}^{2}}$$

## 3. Results and Discussion

### 3.1. Sols and Xerogels

All formulations presented adequate viscosity for application (see Table 2), within the range of pure water and those of other commercial alkoxysilane-based products applied for the same purpose (e.g., current commonly used consolidants’ viscosities: CaLoSiL${}^{®}$E: 1.6–3.0 mPa·s [52]; CaLoSiL${}^{®}$IP: 2.6–3.6 mPa·s [52]; SILRES${}^{®}$BS OH 100: 1.6 mPa·s [53]; TEGOVAKON${}^{®}$V100: 4.5 mPa·s [28,54,55]). They also presented an adequate apparent gelling time (Table 2), and thus, all reactions were presumed to occur within an optimal period upon application. The density of all products was rather similar (Table 2), even upon the addition of modifying agents.

**Figure 1:**
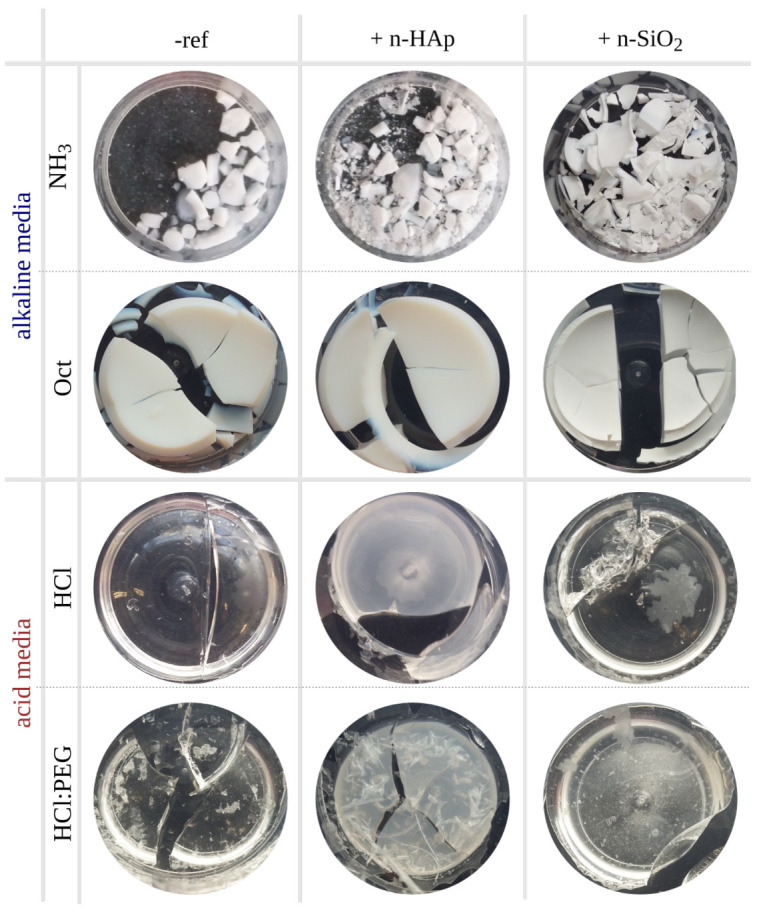
Top view of the xerogels produced from the molar ratios presented in Table 1.

Both a small group of highly cracked and several monolithic xerogels were obtained inside cylindrical flasks (Figure 1). The fact that not all xerogels were completely colourless or without cracking has relative importance, as the authors have observed in previous works [47], since flasks represent a different gelling environment when compared to the stone pores [44], and positive results can still be obtained within the carbonate-medium [45,47].

As previously reported [45], the appearance of xerogels (Figure 1) is clearly different when comparing acidic and basic catalysis: acid catalysis formed transparent xerogels, whereas basic catalysis formed opaque-white ones. The addition of nano-HAp and nano-SiO_2_ as modifiers also contributed to the modification of the appearance of xerogels, in particular to the reduction of transparency in cases such as HCl:nHAp and HCl:PEG:HAp. The cracking degree (visible in the xerogels presented in Figure 1) decreases upon the addition of nano-HAp and nano-SiO_2_ to all acid formulations, but not to alkaline ones.

**Figure 2:**
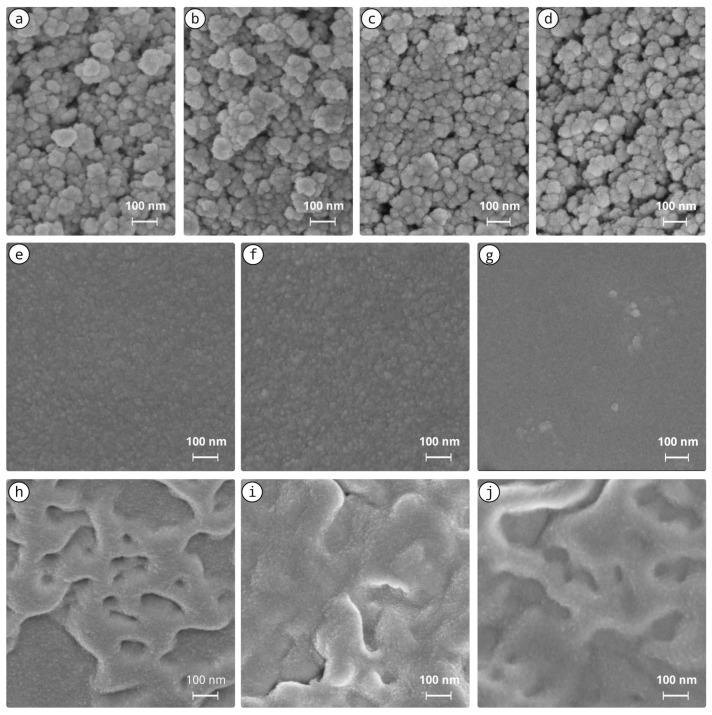
Exemplary scanning electron micrographs of the nanocomposite xerogels produced under alkaline and acid catalysis. (**a**) NH_3_-ref; (**b**) NH_3_:nHAp; (**c**) Oct-ref; (**d**) Oct-nHAp; (**e**) HCl-ref; (**f**) HCl:nHAp; (**g**) HCl:nSiO_2_; (**h**) HCl:PEG-ref; (**i**) HCl:PEG:nHAp; (**j**) HCl:PEG:nSiO_2_.

SEM observations of the microstructures of xerogels are presented in Figure 2: some examples show alkaline sols (a–d), and all acid sols microstructures are shown (e–j). The microstructures of alkaline formulations (NH_3_ and Oct) were composed of larger well-defined silica nano-spheres forming connected networks (e.g., Figure 2a–d), whilst acid-catalysed formulations (HCl) were composed of a well-interconnected silica structure, likely with pores smaller than visible light wavelengths, and hence macro-scale clear (transparent) materials (Figure 1) [56].

When comparing the microstructures of alkaline sols with and without the addition of the modifiers, small differences can be observed (see the examples presented in Figure 2a–d). Hints of silica nanoparticles more or less aligned in agglomerates around 100 nm long seemed to be visible upon HAp addition (e.g., Figure 2d), but the differences were subtle. The possibility that HAp could serve as small nuclei for the growth of silica particles has already been proposed [38]. In acid formulations with no PEG, also mall differences in morphology can be observed (Figure 2d–f). In the case of the HCl:PEG-catalysed sols, some influence on the microstructure can be observed. The surface features, presumably related to the PEG chains added, seemed to become more spread within the structure, which in turn can result in the lower microcracking observed macroscopically in Figure 1. This seemed to have some influence on the nano-SiO_2_ addition, despite not being visible, likely due to their role as nucleation sites. In turn, the presence of nano-HAp was not completely evident in the SEM observations, since it seemed to be well dispersed in the network, but in the two acid—HCl and HCl:PEG—formulations, it clearly promoted a macroscopic light interference effect (whitening of the HCl:nHAp and HCl:PEG:nHAp xerogels).

**Figure 3:**
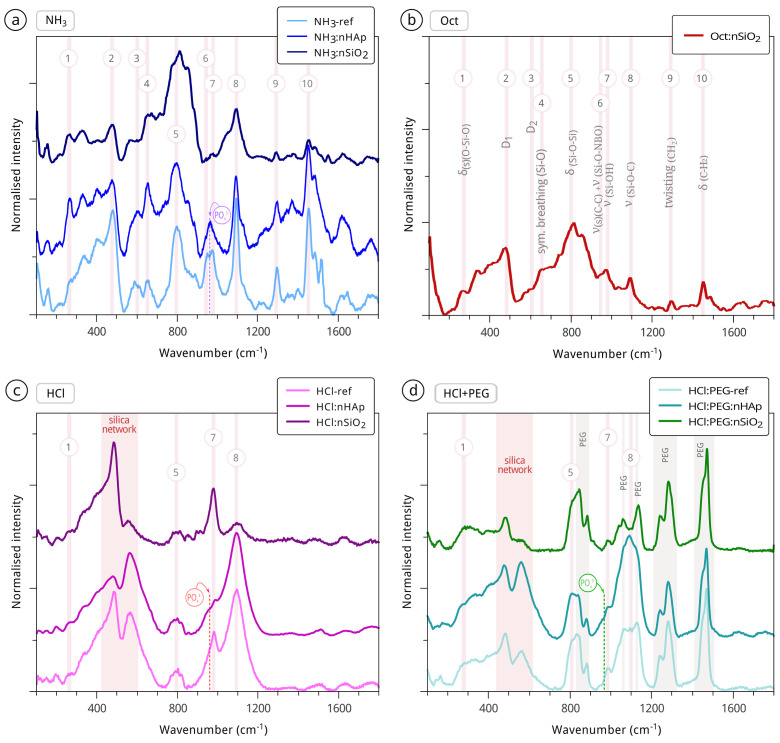
Baseline corrected Raman spectra of the four different series of formulations, catalysed by (**a**) NH_3_, (**b**) Oct, (**c**) HCl, and (**d**) HCl modified with the addition of PEG and identified according to literature data [57,58,59,60,61,62,63].

A deeper understanding of the two types of nanocomposite formulations from the chemical point of view was performed through Raman spectroscopy, the resulting spectra of which are shown in Figure 3. Oct-ref and Oct:nHAp are not able to be presented due to the background fluorescence undermining the interpretation of the nanocomposite Raman bands. In the Figure 3 spectra, it is visible that acid and basic media sols developed different final structures (e.g., Figure 3a,b vs. Figure 3c,d).

The addition of nano-HAp (ν1: P−O of the tetrahedral PO_4_ group vibrating at ca. 961 cm^−1^ [64,65]) resulted in a small modification of the alkaline NH_3_-ref structure. The reference alkaline xerogels (Figure 3a,b) were less hydrolysed, as suggested by the presence of the following Raman bands: (i) strong C−H_2_ vibration modes (marked as 9 and 10); (ii) the visible modes of Si−O in Q${}_{0}$ tetrahedra ca. 654 cm^−1^ (symmetric breathing), and ca. 800 cm^−1^ (symmetric stretching) [58,62]; (iii) TEOS ethoxy groups (−OCH_2_CH_3_ vibration at ca. 400 cm^−1^ [60], marked as 2). The observation of these bands is consistent with the SEM coarser structure of alkaline formulations visible in Figure 2. On the contrary, in the NH_3_:nSiO_2_- and Oct:nSiO_2_-modified formulations, the intense symmetric stretching of Si−O in Q${}_{0}$ tetrahedra peaking around 800 cm^−1^ seemed to be related to the addition of the hydrophilic silica nanoparticles that were incorporated into the final structure.

The addition of nano-HAp to the acid formulations seemed to have contributed to a small reduction of the D_1_ mode (breathing vibration mode of the four membered rings [66]) at around 490 cm^−1^ and the previously mentioned Si−OH stretching mode, whilst the opposite was observed with the addition of the nano-SiO_2_ particles (cf. Figure 3c). This was likely a consequence of the incorporation of the nano-SiO_2_ into the silica network—which is usually a well-interconnected silica network, consistent with the SEM observations (Figure 2), also visible through: (i) the typical peaks in the range of 430–600 cm^−1^ (ca. 500 cm^−1^ broad) of the Si−O−Si mixed stretching and bending modes [59,61]), increased at the expense of the TEOS ethoxy groups’ hydrolysis (displacement of bands from ca. 400 cm^−1^ [63]); (ii) a clear increase of Si−OH modes around 1000 cm^−1^ [58] (marked as 7). In the HCl:PEG:HAp xerogel spectrum (Figure 3d), the PEG CH_2_,C−H and C−O−C modes were visible around ca. 830 and 880 cm^−1^, ca. 1060 cm^−1^, 1130 cm^−1^, and 1460 cm^−1^ and around 1240 cm^−1^ and 1280 cm^−1^, respectively [61,67].

**Figure A2:**
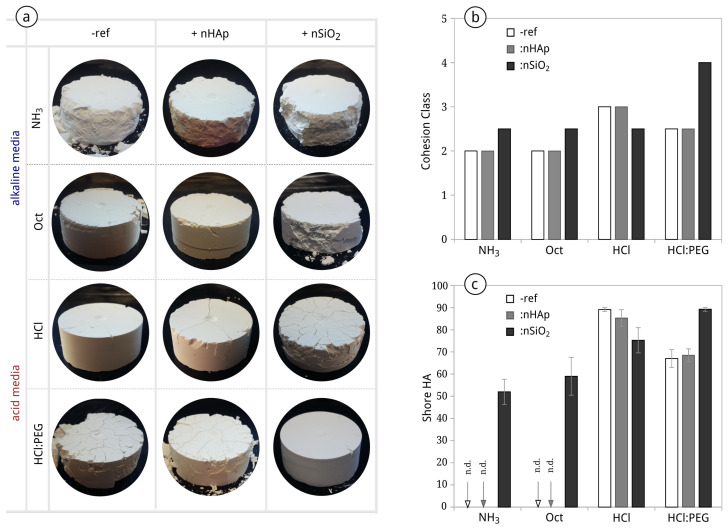
Results obtained from the calcite blends produced with the prepared formulations: (**a**) side view of the blends’ monoliths, (**b**) cohesion class, and (**c**) the Shore A hardness (HA) measurements.

Most formulations promoted the formation of more or less cohesive monoliths, providing potential to increase cohesion (see Appendix B, Figure A2). These products’ ability to promote cohesion was also tested via handling and Shore A hardness (Figure A2)—both tests have been proven to be complementary in the case of different degrees of cohesion increase [45,47].

### 3.2. Potential Initial Efficacy and Compatibility Issues

All reference and modified formulations reached the opposite side of all sound Ançã stone samples when these were treated by capillary suction (30 mm depth)—see Appendix B, Figure A2. The amount of product absorbed by the stone samples, together with the amount of product retained are relevant factors for evaluating the consolidation behaviour [41,68], here from 7–10 kg/m^2^ for all cases. It was verified that independently of the modifiers added to each case, there was a higher dry residue occurring in acid-catalysed formulations than alkaline-catalysed ones (an order of magnitude difference in kg/m^2^; see Table 3 and Table 4)—as observed elsewhere [45].

The efficacy of the nanocomposites was evaluated through the comparison of the mechanical properties of the sound Ançã stone before and after the treatment, as well as by comparison of the effect on those properties of reference (-ref) formulations in relation to nano-HAp- and nano-SiO_2_-modified ones.

**Figure 4:**
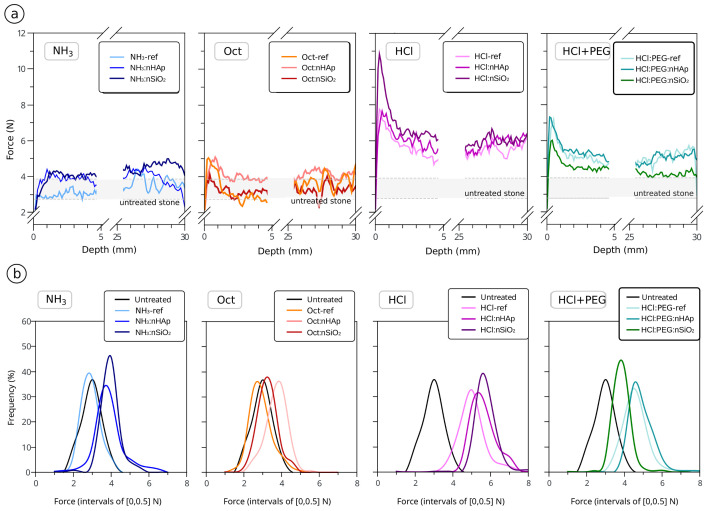
Drilling resistance average profiles (**a**) and respective histograms (**b**): comparison and evaluation of the influence of the prepared formulations on the resistance of treated sound samples, separated by different catalysts, in relation to the untreated stone.

The average drilling resistance (DR) profiles in Figure 4a show that several of the nanocomposite formulations promoted homogeneous in-depth increments in relation to untreated stone. Stone samples treated with alkaline-catalysed formulations seemed to possess a poor strengthening potential (Figure 4), correlated with the much lower dry residue (Table 3 and Table 4) and inferior interconnection of the matrix (as observed in Figure 2).

The addition of nano-HAp to NH_3_-catalysed formulations slightly increased the DR values, whereas the addition of nano-SiO2 only increased the DR in the NH_3_ formulation. Despite the increase observed in some of the alkaline formulations due to the modifiers’ action, the final strengthening ability was still much lower than the ones exhibited by acid-catalysed formulations (with or without modifiers).

In the formulations catalysed by HCl, the addition of nano-HAp and nano-SiO_2_ had a very incipient role, and in the case of HCl:PEG:nSiO_2_, the modifier addition caused an overall loss of the strengthening potential. In the particular case of the HCl:nSiO_2_ nanocomposite, some strength gain occurred, peaking at the surface (i.e., a ∼1–2 mm superficial “crust”). This was followed by an increase in the resistance to drilling in-depth compared to the untreated stone samples. Superficial strength peaks are usually explained by changes in the sol–gel route, which promotes accumulation at the surface or by reverse migration. The latter may be a more likely reason in this scenario, since in all cases, it was verified that the capillary fringe reached the opposite side of the stone samples.

**Figure 5:**
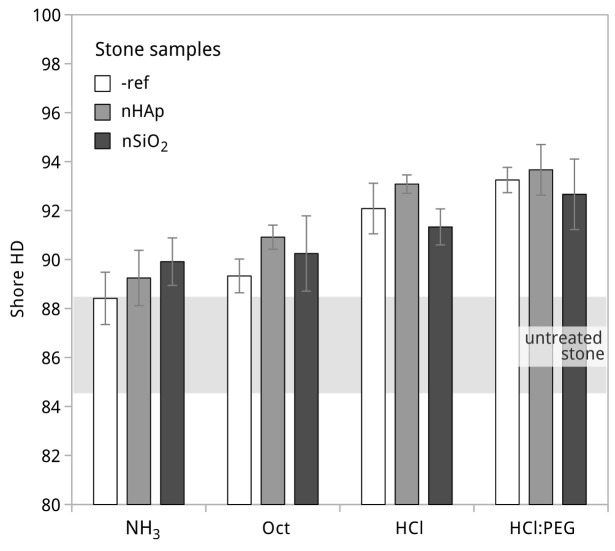
Shore D hardness of the stone surfaces after treatment with all prepared formulations.

The increase of the Shore D hardness of treated surfaces compared to untreated surfaces (Figure 5) did not always correspond to the formation of a hardened superficial layer. In this study, Shore D is a fair indication of in-depth strength gain, well correlated with the results in Figure 4—the only exception being the treatment of samples with the HCl:nSiO_2_ formulation. In the latter, there was in fact the formation of a small 1–2 mm “crust”, which is known to be dependent on the type of treatment procedure, in particular on the one applied here [41,69]. Moreover, the initial increase in strength can be sometimes desirable given certain deterioration profiles of these limestones (e.g., [1]); hence, this matter is further discussed in Section 3.3.

When comparing the reference formulations with the effect of the addition of nano-HAp, it seems clear that both products slightly increased the DR and Shore D hardness in all cases. In the case of the addition of nano-SiO_2_, a strengthening effect was not clear for all cases and was mostly visible in NH_3_:nSiO_2_ and HCl:PEG:nSiO_2_.

The products providing a significant in-depth DR increase (see the histograms in Figure 4b) and achieving a minimal penetration depth of 15–20 mm [12,41,70] were mainly the ones catalysed by HCl (HCl and HCl:PEG series), either reference or modified formulations. All the latter presented in Figure 4 revealed good potential for application, providing a strength increase up to at least a 25 mm depth, and even up to a 30 mm depth in most cases.

**Figure A4:**
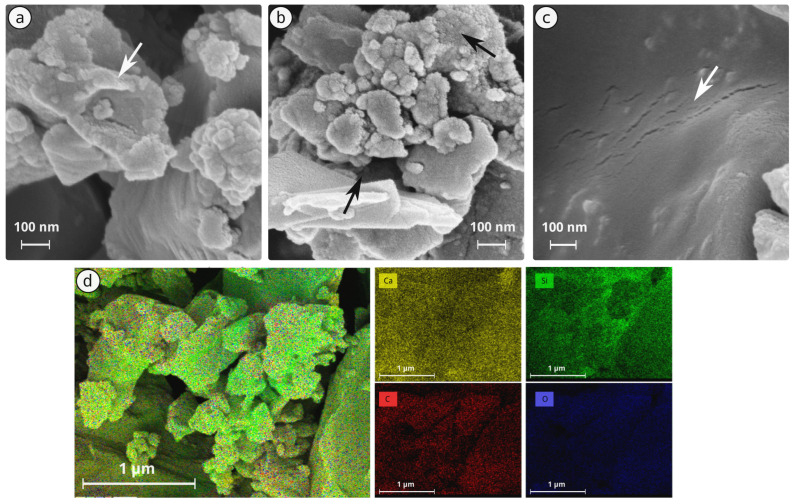
Exemplary SEM micrographs of the stone samples treated with (**a**) HCl-ref, (**b**) HCl:nHAp, and (**c**) HCl:nSiO_2_ and (**d**) exemplary EDS results from one of the samples treated with HCl-ref formulation. Arrows indicate the areas where a film covering calcite grains and stone pores is visible or was analysed.

Through the SEM observations (see Appendix D, Figure A4), it was shown that the treatments tested and having better potential formed a film that bridged the grains and filled the grooves present in the stone.

**Figure 6:**
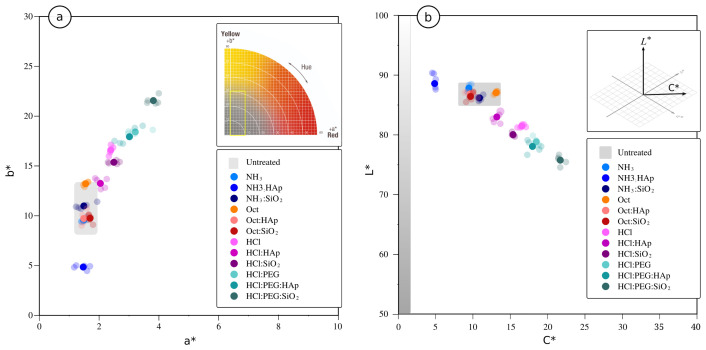
Colour variation of the stone surfaces upon treatment with all prepared formulations: (**a**) a* vs. b* and (**b**) C* vs. L* parameters.

The treated samples were submitted to colourimetric characterisation for the evaluation of the colour alterations, which can be a key indicator when selecting one treatment over another with similar effectiveness. Colour changes determined caused by the treatments on the stone surfaces are presented in Figure 6 and Table 5. As can be observed by the dispersion in Figure 6a,b and from the ΔE* values in Table 5, the samples treated with alkaline-catalysed (NH_3_ or Oct) formulations revealed low to medium levels of colour variations, whilst acid-catalysed (HCl and HCl:PEG) formulations showed the greatest changes. Nonetheless, the HCl:nHAp sol caused moderate colour alterations (ΔE* <5) compared to the HCl-ref sol, where the ΔE* value can already be considered a high incompatibility risk [12]. The darkening (reduction in L*) and yellowing (increase of b*) of the surfaces treated with the later product were the lowest among all acid-catalysed formulations, when comparing all values of the treated surfaces with those of the untreated samples (see Figure 6a,b). Ançã stone is a very light-coloured and homogeneous stone, and its colour is particularly sensitive to conservation treatments [5]. Additionally, upon ageing, it tends to possess a yellowish tone [1]. Variations among sound samples can go up to values of ΔE* = 1.9. The HCl-catalysed formulations, especially those with the addition of PEG, promote a “wet-surface” visual effect, reducing the luminosity (L*) and changing the chroma (C*—Figure 6b) to a yellowish hue (Figure 6a). As was elsewhere mentioned, it often occurs that the increase in effectiveness (i.e., strengthening) is made at the expense of an increase in harmfulness [1]. When comparing the low potential of consolidation of the alkaline-route solutions (NH_3_- and Oct-catalysed formulations with any of the modifiers added), the acid-catalysed formulations seem more promising. A balance among effectiveness, colour alteration, and other compatibility aspects not addressed here needs to be considered [1].

### 3.3. Treatment Trials to Reduce the Risk of Incompatibility

Considering the balance between the DR and colour alterations obtained in the previous section, two of the formulated nanocomposites—with evident potential to increase the DR of carbonate stones and the smallest colour alterations possible—were selected for further treatments trials: HCl:nHAp and HCl:nSiO_2_.

The two showed some tendency to produce a “crust” and some colour alteration when applied by a capillary absorption, which allows a constant and continuous flow supply of the product. Different application procedures have been reported to influence the absorption of products and on the subsequent consolidation of the substrates [41], which may need to be considered when studying the effects of modifying a certain product. Thus, the evaluation of the possible influence of the treatment procedure type and stone support characteristics may be of interest. HCl:nHAp and HCl:nSiO_2_ were applied into Ançã stone samples (sound and aged samples) by brushing, which is a common application procedure used in conservation practise [5]. The amount of product retained by the samples is shown in Table 6 for both brushing and capillary absorption treatments.

The brushing allowed the application of relatively similar amounts of product when compared to capillary absorption, and the condition of the stone seemed to have little impact on its capacity to absorb the product. The small differences in absorbed product (see % dry residue in Table 6) seemed to be of no relevance for the final amount of product retained within the stone pores, which was similar (in kg/m^2^, Table 6) in the stones treated with HCl:nHAp and HCl:nSiO_2_, independent of the application method or stone condition.

**Figure 7:**
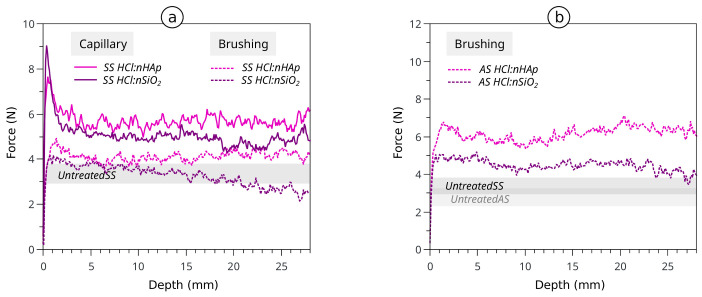
Drilling resistance average profiles of the treatment by brushing with HCl:nHAp and HCl:nSiO_2_ on (**a**) sound stone (SS) and (**b**) aged stone (AS) samples.

The drilling profiles for the sound and aged stone samples treated with HCl:nHAp by brushing are shown in Figure 7. It is clearly visible that in all cases, there was an increase of the average drilling resistance upon treatment with this modified formulation. When comparing the behaviour of HCl:nHAp with the one of HCl:nSiO_2_, it became clear that the strength increase was significant in HCl:nHAp for all the circumstances.

Although the amounts of product applied were similar, the efficacy of the formulations was revealed to be fairly dependent on the type of treatment procedure. It is interesting to note that the brushing procedure with both formulations seemed to: (a) increase the resistance of treated stones to lower values when compared to the ones treated with the same formulation by capillarity absorption, (b) have a lesser tendency to develop superficial “crusts”, but also (c) reduce the penetration of the products in-depth, for instance in the case of sound and aged stones treated with HCl:nSiO_2_ (Figure 7).

A summary of the results of the initial assessment performed on the two treatments with the two selected nanocomposite formulations, by both capillary suction and brushing procedures, is presented in Table 7. These results translate the presence of higher DR increments correlated with “crust” formation and significant colour alteration mainly with the use of the capillary absorption procedure, whilst lower increments were registered for brushing, where the hardened superficial layer seemed to be absent and the colour alterations were less significant. Colour alteration was reduced and some strengthening was observed with the brushing treatment with HCl:nSiO_2_, and a good compromise of effectiveness vs. compatibility or effectiveness vs. harmfulness seemed to be observed with the novel HCl:nHAp nanocomposite studied here.

## 4. Conclusions

The potential of a set of novel formulations was investigated for the consolidation of soft carbonate stone through the modification of acid and alkaline TEOS-based formulations with newly developed HAp nanorods and nano-SiO_2_. Potential improvements on efficacy and moderate colour alterations were achieved with both types of modifiers. The NH_3_- and Oct-catalysed formulations (alkaline-route solutions) showed a lower potential of consolidation with any of the modifiers added compared to the acid-catalysed formulations. Nonetheless, the former showed also lower harmfulness than the latter (e.g., colour alteration). The increase in stone cohesion, adequate in-depth penetration patterns, as well as colour compatibility with the substrate was obtained with several formulations through two types of treatment (capillary absorption and brushing), both in sound and aged stone samples. After an initial assessment and taking into consideration the aforementioned variables, the modification of TEOS-based formulations, inclusive of the newly developed HAp nanorods, revealed interesting results to be further examined. The presented results obtained with the modification of the studied set of alkoxysilane-based formulations with either nano-SiO_2_ and nano-HAp encourage further research on the influence of the type of particles and catalyst focusing, for instance on the amount of incorporation of particles, the influence of the catalyst, and/or the application process of these modified products. The current and further research findings may be of practical interest to several scientific disciplines that still actively work on finding the the most adequate materials to be applied whilst tackling the many unsolved problems of the conservation of carbonate stone built heritage.

## Figures and Tables

**Table 1:** Molar ratios of the several formulations prepared in the present work: TEOS:EtOH:Catalyst:H_2_O:PEG.

TEOS	EtOH	Catalyst		H_2_O	PEG Added	Designation	Modifier (1:0.001)	Designation
1	7.6	HCl	down to pH’ = 3.2	2.1	—	HCl-ref	nano-HAp	HCl:nHAp
							nano-SiO_2_	HCl:nSiO_2_
					0.11	HCl:PEG-ref	nano-HAp	HCl:PEG:nHAp
							nano-SiO_2_	HCl:PEG:nSiO_2_
		Oct	0.002	1.5	—	Oct-ref	nano-HAp	Oct:nHAp
						Oct-ref	nano-SiO_2_	Oct:nSiO_2_
		NH_3_	0.008		—	NH_3_-ref	nano-HAp	NH_3_:nHAp
						NH_3_-ref	nano-SiO_2_	NH_3_:nSiO_2_

**Table 2:** Characterisation of the formulations’ properties: viscosity (mPa·s) at 24.5 °C, approximate gelling time (in d), and density (g/mL).

Sols Characteristics									
	Viscosity (mPa·s)			Gelling Time (d)			Density (g/mL)		
	-ref	nHAp	nSiO_2_	-ref	nHAp	nSiO_2_	-ref	nHAp	nSiO_2_
NH_3_	1.1	1.0	1.1	13d	13d	13d	0.83	0.84	0.84
Oct	0.9	0.8	0.9	6d	6d	6d	0.84	0.84	0.85
HCl	1.7	1.7	1.6	14d	14d	14d	0.83	0.84	0.85
HCl:PEG	1.9	1.8	2.0	13d	13d	13d	0.87	0.86	0.86

**Table 3:** Final dry residue (%) obtained in the three different drying environments (±1%).

Dry Residue (%)									
	Xerogels			Calcite Blends			Stone Samples		
	-ref	nHAp	nSiO_2_	-ref	nHAp	nSiO_2_	-ref	nHAp	nSiO_2_
NH_3_	2%	2%	4%	3%	3%	6%	1%	1%	2%
Oct	12%	12%	7%	11%	11%	10%	2%	2%	2%
HCl	14%	14%	16%	12%	12%	14%	15%	13%	18%
HCl:PEG	19%	19%	19%	19%	20%	19%	25%	20%	20%

**Table 4:** Absorbed product and dry residue after the treatment procedure on the stone specimens (in kg/m^2^).

Units: kg/m^2^	Absorbed Product			Dry Residue		
	-ref	nHAp	nSiO_2_	-ref	nHAp	nSiO_2_
NH_3_	7.48	7.50	8.16	0.06	0.08	0.15
Oct	9.21	8.56	10.17	0.15	0.18	0.14
HCl	8.66	8.66	8.35	1.14	1.05	1.37
HCl:PEG	7.31	7.87	8.38	1.65	1.42	1.48

**Table 5:** Overall total colour change (ΔE*) after consolidation treatment by comparison with untreated surfaces.

Total Colour Variation (ΔE*)							
	Alkaline				Acid		
	ref	nHAp	nSiO_2_		ref	nHAp	nSiO_2_
blackNH_3_	1.6	5.1	1.1	HCl	8.4	4.8	8.4
Oct	3.4	0.6	0.4	HCl:PEG	11.5	11.7	16.0

**Table 6:** Final dry residues measured on treated sound stone (SS) and aged stone (AS) samples.

	Dry Residue (%)			(kg/m^2^)
	SS Capillary	SS Brushing	AS Brushing	All Cases
HCl:nHAp	13%	17%	17%	1.0±0.0
HCl:nSiO_2_	18%	18%	18%	1.2±0.2

**Table 7:** Summary of the parameters assessed for sound stone (SS) and aged stone (AS) samples treated with HCl:nHAp and HCl:nSiO_2_ by capillary suction and brushing. Legend: DR ↑—drilling resistance increment; Depth—maximum penetration depth (in mm) observed through DR measurements; HD—Shore hardness D; ΔE*—total colour variation.

	SS Capillary Abs.				SS Brushing				AS Brushing			
	DR↑	Depth	HD	ΔE*	DR↑	Depth	HD	ΔE*	DR↑	Depth	HD	ΔE*
HCl:nHAp	62%	30	93.1	4.8	22%	30	87.9	1.3	55%	30	90.7	2.0
HCl:nSiO_2_	73%	30	91.3	8.4	15%	15	89.4	2.4	37%	20	90.4	4.3

**Figure A1:**
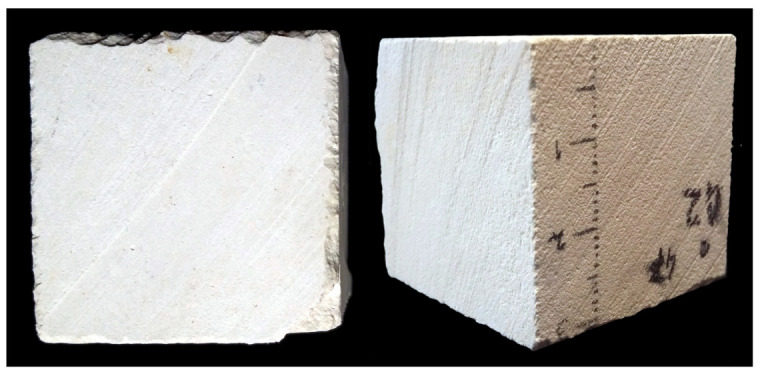
Example of the Ançã stone samples used for this study.

**Figure A3:**
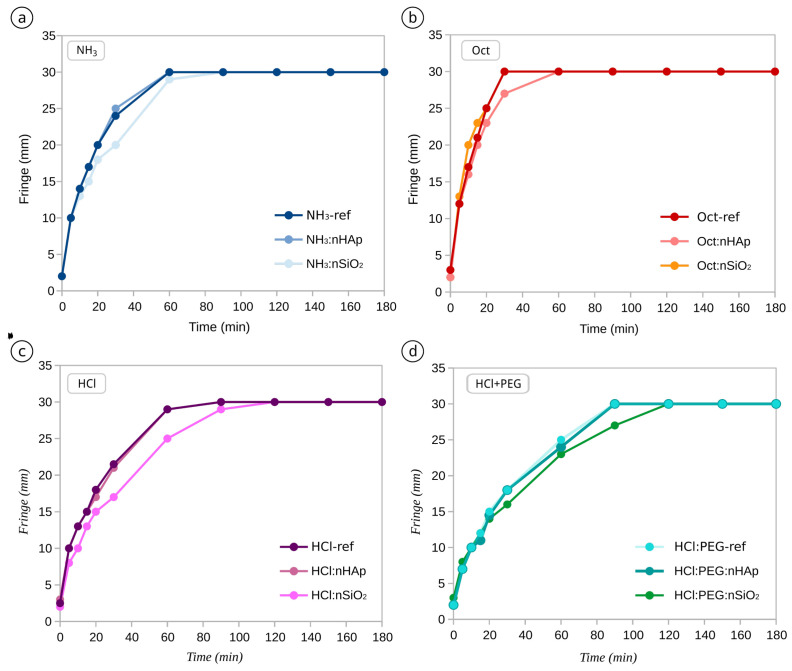
Capillary suction fringe evolution upon treatment with all the formulations under study.

## Appendix B. Calcite Blends

To better understand the possible results within the carbonate medium and the potential for the cohesion increase of the two types of nanocomposites, the produced formulations were blended with CaCO_3_ powder. Most formulations promoted the formation of more or less cohesive monoliths of calcite blends, as can be observed in Figure A2.

The preliminary assessment of these products’ ability to promote cohesion was performed via handling and Shore A hardness (Figure A2b,c). Both tests have been proven to be complementary [45,47], since the expedite empirical test presents itself as a good indication in cases with lower cohesion, such as in the case of monoliths of blends where Shore A hardness measurements are not able to be obtained (see Figure A2c). The results presented in Figure A2a–c show that the addition of nano-SiO_2_ to alkaline formulations (NH_3_ and Oct) and to acid formulations with the addition of PEG promoted an apparent cohesion increase compared to the reference formulations, whilst an increase upon the addition of nano-HAp was clearly visible with this test. Interestingly, the modification with nano-SiO_2_ seemed to promote an apparent cohesion increase, and all products were tested envisioning these comparative purposes (i.e., reference vs. nano-HAp- vs. nano-SiO_2_-modified formulations).

## Appendix D. SEM after Stone Treatment (Supplementary)

Through the SEM observations shown in Figure A4 (some examples for the HCl-catalysed formulations were selected), it is visible that the treatments tested with better potential formed a film that bridged the grains and filled the grooves present in the stone. Through the examples given in Figure A4a–c, the following are visible: (a) film over the calcite grains, (b) film over and on the interstices of the stone, and (c) film covering the stone surface—and these observations were attested by the EDS distribution of the silica-based material in Figure A4d. The latter was widespread and filled the stone pores observed in the micrometric scale, showing the ability of the prepared formulations and treatments to cover the substrate.
